# Maternal Vulnerability Index and Severe Maternal Morbidity

**DOI:** 10.1001/jamanetworkopen.2025.17068

**Published:** 2025-06-23

**Authors:** Nansi S. Boghossian, Joshua Radack, Molly Passarella, Ciaran S. Phibbs, Lucy T. Greenberg, Jeffrey S. Buzas, George R. Saade, Jeannette Rogowski, Scott A. Lorch

**Affiliations:** 1 Department of Epidemiology and Biostatistics, Arnold School of Public Health, University of South Carolina, Columbia; 2 Leonard Davis Institute of Health Economics, Wharton School, University of Pennsylvania, Philadelphia; 3 Health Economics Resource Center and Center for Implementation to Innovation, Veterans Affairs Palo Alto Health Care System, Menlo Park, California; 4 Departments of Pediatrics and Health Policy, Stanford University School of Medicine, Stanford, California; 5 Vermont Oxford Network, Burlington, Vermont; 6 Department of Mathematics and Statistics, University of Vermont, Burlington; 7 Department of Obstetrics and Gynecology, Eastern Virginia Medical School, Norfolk; 8 Department of Health Policy and Administration, The Pennsylvania State University, State College; 9 Department of Pediatrics, University of Pennsylvania School of Medicine, Philadelphia

## Abstract

**Importance:**

Few studies have investigated the association of composite measures of neighborhood social determinants of health with severe maternal morbidity (SMM), and no research has examined this association for indices tailored to maternal health.

**Objective:**

To examine the association of scores in the Maternal Vulnerability Index (MVI), a tool developed to measure maternal risk of adverse health outcomes, with SMM.

**Design, Setting, and Participants:**

This retrospective, population-based cohort study was conducted in 5 states (2008-2020 for Michigan, Oregon, and South Carolina; 2008-2018 for Pennsylvania; and 2008-2012 for California) among individuals delivering a fetal death or a live birth between 22 and 44 weeks. Analysis was conducted between August and October 2024.

**Exposure:**

The MVI, a composite measure of 43 area-level indicators, was categorized into 6 themes encompassing physical, social, and health care environments. MVI score and themes were examined in quartiles (quartile 1 = lowest risk to quartile 4 = highest risk) based on residential zip code tabulation area.

**Main Outcomes and Measures:**

SMM during delivery hospitalization and after discharge within 42 days after delivery.

**Results:**

Among 6 543 255 birthing individuals (3 568 631 ages 25-34 years [54.5%]; 472 145 Asian or Pacific Islander [7.2%], 824 239 Black [12.6%], 1 673 917 Hispanic [25.6%], and 3 346 807 White [51.2%]), there were 1 087 936 individuals in MVI quartile 1 (16.6%) and 1 376 658 individuals in MVI quartile 4 (21.0%). A total of 45 051 individuals (0.7%) had SMM during delivery hospitalization, while 13 534 individuals (0.2%) had SMM after discharge within 42 days after delivery. In adjusted analyses, there were no associations between MVI score or themes and SMM during delivery hospitalization. However, a dose-response association was observed between MVI score and SMM within 42 days after delivery (second MVI quartile: adjusted relative risk [aRR], 1.03; 95% CI, 0.95-1.11; third MVI quartile: aRR, 1.12; 95% CI, 1.03-1.23; fourth MVI quartile: aRR, 1.27; 95% CI, 1.14-1.41). The highest MVI quartile in themes of general health care (aRR, 1.27; 95% CI, 1.14-1.43), physical environment (aRR, 1.33; 95% CI, 1.22-1.46), physical health (aRR, 1.23; 95% CI, 1.12-1.35), reproductive health care (aRR, 1.30; 95% CI, 1.15-1.47), and socioeconomic determinants (aRR, 1.19; 95% CI, 1.02-1.39) was associated with SMM within 42 days after delivery. A dose-response association was observed between all MVI themes and SMM within 42 days after delivery (eg, physical environment MVI theme second quartile: aRR, 1.04; 95% CI, 0.96-1.13; third quartile: aRR, 1.14; 95% CI, 1.05-1.25; fourth quartile: aRR, 1.33; 95% CI, 1.22-1.46), except for the mental health and general health care themes.

**Conclusions and Relevance:**

In this study, MVI score was not associated with SMM during delivery but was associated with postpartum SMM, suggesting that MVI may capture long-term risks more effectively than acute conditions during delivery hospitalization.

## Key Points

**Question:**

Is the score on the Maternal Vulnerability Index (MVI), a tool to measure maternal risk of adverse health outcomes, associated with severe maternal morbidity (SMM)?

**Findings:**

In this cohort study of 6 543 255 birthing individuals from 5 states, a dose-response association was observed between MVI quartile and SMM within 42 days after delivery but not for SMM during delivery hospitalization.

**Meaning:**

These findings suggest that the MVI may capture long-term risks more effectively than acute conditions seen during delivery hospitalization.

## Teaser

This cohort study investigates the association of the Maternal Vulnerability Index score, a composite measure of area-level indicators including physical, social, and health care environments, with severe maternal morbidity during delivery hospitalization and within 42 days of birth.

## Introduction

The US has the highest maternal mortality rate among 11 developed countries.^1^ Even more extreme are the rates of severe maternal morbidity (SMM); for every maternal death, more than 100 birthing individuals experience an SMM event.^2^ Previous research on SMM has primarily focused on individual-level factors, such as race and ethnicity,^3,4,5,6^ gestational weight gain,^7^ prepregnancy obesity,^4,8^ comorbidities,^3,4,6^ and socioeconomic status.^3,4^ Few studies have explored broader factors, such as the quality of obstetric care^9,10,11,12^ and neighborhood-level influences. Examples of neighborhood-level factors examined in association with SMM include poverty,^4,6,12,13^ crime,^6^ racial distribution,^6^ racial inequities in female educational attainment and employment,^14^ housing affordability,^15^ and racial and economic spatial polarization.^16^ While 4 studies^11,17,18,19^ have investigated the association of composite measures of neighborhood social determinants of health with SMM, to our knowledge, no research has specifically examined this association for neighborhood- or community-level indices tailored to maternal health. Such information is critical for policymakers to accurately identify at-risk populations, design targeted interventions, and effectively address the maternal health crisis.

In 2023, Surgo Health developed the Maternal Vulnerability Index (MVI), a county-level composite metric encompassing 43 indicators identified as key drivers of maternal health outcomes. These indicators were grouped into 6 themes: (1) reproductive health care, (2) physical health, (3) mental health and substance use, (4) general health care, (5) socioeconomic determinants, and (6) physical environment.^20^ Studies indicate that higher MVI scores in a mother’s residential county are associated with increased odds of maternal mortality, low birthweight, preterm births, and infant mortality.^20,21,22^ We conducted a retrospective cohort study to examine whether scores for the overall MVI and its 6 underlying themes for a mother’s residential zip code were associated with SMM.

## Methods

### Study Sample

In this cohort study, we developed a deidentified cohort of deliveries using birth and fetal death certificates with gestational age between 22 and 44 weeks linked to maternal and infant inpatient hospital discharge records, as previously described,^23^ from California, Michigan, Oregon, Pennsylvania, and South Carolina. The study period included 2008 to 2020 for Michigan, Oregon, and South Carolina; 2008 to 2018 for Pennsylvania; and 2008 to 2012 for California. Delivery and inpatient hospitalizations up to 42 days after delivery were available for all 5 states. Additionally, we used inpatient hospitalization data during pregnancy and postdelivery discharge extending through 365 days after delivery for Michigan, Oregon, and South Carolina. State health departments provided the linked data after removing any identifying information. The institutional review boards of the Children’s Hospital of Philadelphia, University of South Carolina, and departments of health in the 5 states approved the study. Informed consent was not obtained for this study because all records were deidentified. We followed the Strengthening the Reporting of Observational Studies in Epidemiology (STROBE) reporting guideline. Analysis was conducted between August and October 2024.

### Study Variables

The primary outcomes were SMM during delivery hospitalization and SMM after discharge within 42 days after delivery. SMM was defined using the updated Centers for Disease Control and Prevention (CDC) *International Classification of Diseases, Ninth Revision* (*ICD-9*) or *International Statistical Classification of Diseases and Related Health Problems, Tenth Revision* (*ICD-10*) diagnosis and procedure code algorithms,^24^ which encompass 16 life-threatening maternal conditions (eg, heart failure, kidney failure, and sepsis) and 4 lifesaving procedures (eg, hysterectomy and ventilation), excluding blood transfusion.^24^ To bridge the transition from *ICD-9* to *ICD-10*, we referenced the Maternal and Child Health Bureau’s federally available data resource document.^25^ Secondary outcomes included readmission within 42 days after delivery. Other secondary outcomes available only for Michigan, Oregon, and South Carolina included SMM from pregnancy through 42 days after delivery, SMM from pregnancy through 365 days after delivery, SMM occurring only during the postpartum period within 365 days after delivery, and readmission within 365 days after delivery.

Through a data use agreement with Surgo Health, we acquired MVI data for the 2020 zip code tabulation areas (ZCTAs). Developed by the US Census Bureau, ZCTAs serve as area-level equivalents to zip codes. Maternal residential zip codes were converted to ZCTAs using crosswalks available on the UDS Mapper website. The primary exposure was MVI score at the ZCTA level, while secondary exposures included the 6 underlying themes of the MVI, also assessed at the ZCTA level. All exposures were analyzed in quartiles based on the birthing individual’s residential ZCTA, with quartile 1 representing the lowest risk and quartile 4 the highest risk. The methodology for developing the MVI and its 6 themes is described elsewhere.^20^ Briefly, 43 indicators identified as being associated with maternal mortality, morbidity, or both in the US based on a scoping review from 2000 to 2020 were grouped into 6 themes.^20^ Data on women of childbearing age at state and county levels were prioritized over data for other populations and other geographic demarcations.^20^ More recent data sources, such as the Census Bureau American Community Survey, National Survey on Drug Use and Health, and CDC were used to calculate overall MVI and thematic MVI scores.^20^ Factors with consistent associations identified in publicly available datasets were included, and their relevance was further validated through consultation with subject matter experts.^20^ Each geographic unit (state, county, and census tract) was subsequently ranked on a scale of 0 to 100 for each theme and the overall MVI, where 0 represents the lowest risk and 100 the highest risk of adverse maternal health outcomes.^20^ See eTable 1 in Supplement 1 for themes, subthemes, and indicators that the MVI comprises.

Maternal race and ethnicity on the birth or fetal death certificate was based on self-identification and was categorized into Asian or Pacific Islander, non-Hispanic Black (hereafter, *Black*), Hispanic, non-Hispanic White (hereafter, *White*), multiracial, and other. The other race and ethnicity group included birthing individuals whose race was recorded as other on the birth certificate, as well as those identified as American Indian or Alaska Native. Race and ethnicity data were collected from different items; individuals who identified as Hispanic were categorized as Hispanic regardless of their reported race, and race data were not used for those who answered *yes* to Hispanic ethnicity. Multiracial was a self-identified category on the birth certificate, except in South Carolina, where it was not an available option. Race and ethnicity were assessed given their association with the exposure and outcome. Examined covariates included maternal race and ethnicity, type of insurance (private, Medicaid, self-pay, and other), education (no high school, some high school, high school or General Educational Development [GED] test, some college, 4-year college, and >4-year college), birth year, prepregnancy body mass index (calculated as weight in kilograms divided by height in meters squared) category (underweight [<18.5], normal [18.5-24.9], overweight [25.0-29.9], and obesity [≥30]), and an obstetric comorbidity index score.^26^ The obstetric comorbidity index score used a previously validated score that included 27 patient-level risk factors associated with SMM (eTable 2 in Supplement 1).^26^

### Statistical Analysis

We first conducted descriptive analyses examining the distribution of maternal characteristics by MVI quartile. We then examined associations between MVI quartile and SMM using modified Poisson regression models^27^ to estimate unadjusted and adjusted relative risks (aRRs) and 95% CIs with clustered standard errors by hospital. Models were adjusted for race and ethnicity, insurance type, education, birth year, and the obstetric comorbidity index.^26^ We also explored in separate regression models unadjusted and adjusted associations between each MVI theme and SMM. Statistical analyses were conducted using SAS statistical software version 9.4 (SAS Institute).

We conducted 3 sensitivity analyses. The first analysis did not adjust for race and ethnicity owing to the potential collinearity between MVI indicators and measures of structural racism. In the second analysis, we did not adjust for the obstetric comorbidity index given that neighborhood-level determinants of health may be associated with chronic disease. Adjusting for these variables could lead to overadjustment for important associational pathways between MVI and the risk of SMM and readmission. In the third analysis, we restricted the study period to 2016 to 2020 given that the MVI was developed based on data sources during this general time.

## Results

### Study Sample

From the original study sample of 6 892 182 live births, we excluded 294 211 deliveries (4.3%) due to the inability to link the birth certificate to the maternal hospital discharge data. We subsequently excluded an additional 54 716 deliveries (0.8%) due to implausible gestational age or birth weight, missing county of residence, or missing MVI score, resulting in 6 543 255 birthing individuals (3 568 631 ages 25-34 years [54.5%]; 472 145 Asian or Pacific Islander [7.2%], 824 239 Black [12.6%], 1 673 917 Hispanic [25.6%], and 3 346 807 White [51.2%], 134 026 multiracial [2.1%], and 80 127 other race or ethnicity [1.2%]). There were 2 528 774 individuals (38.6%) from California, 1 432 662 individuals (21.9%) from Michigan, 536 358 individuals (8.2%) from Oregon, 1 394 668 individuals (21.3%) from Pennsylvania, and 650 793 individuals (9.9%) from South Carolina. A total of 1 087 936 birthing individuals (16.6%) were in MVI quartile 1 (lowest risk), while 1 376 658 birthing individuals (21.0%) were in quartile 4 (highest risk). In bivariate analyses, several covariates were associated with increased MVI, constituting a greater proportion of the highest vs the lowest quartile; these included Black race (479 902 individuals [34.9%] vs 28 356 individuals [2.6%]), maternal age younger than 20 years (167 039 individuals [12.1%] vs 31 459 [2.9%]), Medicaid insurance (931 446 individuals [67.7%] vs 239 005 individuals [22.0%]), less than a high school diploma (361 059 individuals [26.6%] vs 72 208 individuals [6.9%]), inadequate prenatal care (244 304 individuals [18.8%] vs 65 746 individuals [6.2%]), non-nulliparous status (624 476 individuals [57.4%] vs 879 303 individuals [63.9%], and obesity (411 950 individuals [31.6%] vs 156 286 individuals [15.0%]). Rates of SMM during delivery hospitalization, after discharge within 42 days, and after discharge within 365 days after delivery, as well as readmission within 42 and 365 days after delivery, were all higher among birthing individuals in the highest MVI quartile (Table). For example, SMM rates for the highest vs lowest MVI quartile were 11 614 individuals (0.8%) vs 6873 individuals (0.6%) during hospitalization, 4494 individuals (0.3%) vs 1601 individuals (0.1%) after discharge within 42 days after delivery, and 9066 individuals (0.7%) vs 2741 individuals (0.3%) after discharge within 365 days after delivery. A total of 45 051 individuals (0.7%) had SMM during delivery hospitalization, while 13 534 individuals (0.2%) had SMM after discharge within 42 days after delivery.

**Table.** Maternal Characteristics of Deliveries

Characteristic	Birthing individuals, No. (%)				
	Total (N = 6 543 255)	MVI quartile 1 (n = 1 087 936)	MVI quartile 2 (n = 2 019 547)	MVI quartile 3 (n = 2 059 114)	MVI quartile 4 (n = 1 376 658)
Race and ethnicity					
Asian or Pacific Islander	472 145 (7.2)	174 367 (16.1)	175 259 (8.7)	91 235 (4.4)	31 284 (2.3)
Black	824 239 (12.6)	28 356 (2.6)	102 738 (5.1)	213 243 (10.4)	479 902 (34.9)
Hispanic	1 673 917 (25.6)	179 109 (16.5)	505 435 (25.1)	664 666 (32.3)	324 707 (23.6)
White	3 346 807 (51.2)	674 931 (62.1)	1 170 458 (58.1)	1 017 024 (49.5)	484 394 (35.3)
Multiracial	134 026 (2.1)	23 491 (2.2)	44 932 (2.2)	43 834 (2.1)	21 769 (1.6)
Other	80 127 (1.2)	5988 (0.6)	17 308 (0.9)	25 386 (1.2)	31 445 (2.3)
Missing	11 994	1694	3417	3726	3157
Age, y					
<20	497 304 (7.6)	31 459 (2.9)	116 566 (5.8)	182 240 (8.9)	167 039 (12.1)
20-24	1 429 124 (21.8)	116 077 (10.7)	374 131 (18.5)	518 828 (25.2)	420 088 (30.5)
25-34	3 568 631 (54.5)	657 544 (60.4)	1 170 909 (58.0)	1 091 774 (53.0)	648 404 (47.1)
35-40	845 905 (12.9)	225 945 (20.8)	290 259 (14.4)	215 727 (10.5)	113 974 (8.3)
≥40	202 291 (3.1)	56 911 (5.2)	67 682 (3.4)	50 545 (2.5)	27 153 (2.0)
Payor					
Private	3 388 350 (51.8)	832 960 (76.6)	1 211 878 (60.0)	920 021 (44.7)	423 491 (30.8)
Medicaid	3 034 730 (46.4)	239 005 (22.0)	764 455 (37.9)	1 099 824 (53.4)	931 446 (67.7)
Self-pay	92 480 (1.4)	11 669 (1.1)	32 583 (1.6)	30 121 (1.5)	18 107 (1.3)
Other	27 692 (0.4)	4302 (0.4)	10 630 (0.5)	9146 (0.4)	3614 (0.3)
Missing	3	0	1	2	0
Education					
No high school	275 542 (4.3)	21 706 (2.1)	75 819 (3.8)	104 868 (5.2)	73 149 (5.4)
Some high school	845 182 (13.2)	50 502 (4.8)	191 592 (9.7)	315 178 (15.5)	287 910 (21.2)
High school or GED	1 656 941 (25.8)	150 952 (14.4)	454 610 (22.9)	602 337 (29.7)	449 042 (33.0)
At least some college	1 815 504 (28.2)	235 219 (22.4)	576 373 (29.0)	610 367 (30.1)	393 545 (29.0)
4-y College	1 162 493 (18.1)	348 070 (33.1)	437 632 (22.0)	269 904 (13.3)	106 887 (7.9)
>4-y College	671 092 (10.4)	245 219 (23.3)	249 239 (12.6)	127 980 (6.3)	48 654 (3.6)
Missing	116 501	36 268	34 282	28 480	17 471
Kotelchuck index					
Inadequate	718 882 (11.5)	65 746 (6.2)	172 311 (8.9)	236 521 (12.0)	244 304 (18.8)
Intermediate	790 536 (12.6)	154 081 (14.6)	246 244 (12.7)	231 888 (11.8)	158 323 (12.2)
Adequate	2 656 822 (42.4)	485 952 (46.1)	892 322 (46.1)	847 701 (43.1)	430 847 (33.1)
>Adequate	2 093 289 (33.4)	348 679 (33.1)	624 631 (32.3)	650 544 (33.1)	469 435 (36.0)
Missing	283 726	33 478	84 039	92 460	73 749
Nulliparous	2 582 103 (39.5)	463 460 (42.6)	831 049 (41.2)	790 239 (38.4)	497 355 (36.1)
Cesarean delivery	2 138 847 (32.7)	354 293 (32.6)	657 202 (32.5)	680 895 (33.1)	446 457 (32.4)
Multiple gestation					
Yes	223 588 (3.4)	47 355 (4.4)	69 730 (3.5)	62 352 (3.0)	44 151 (3.2)
Missing	533	77	160	153	143
Prepregnancy BMI					
Underweight (<18.5)	231 321 (3.7)	42 687 (4.1)	70 490 (3.7)	68 759 (3.5)	49 385 (3.8)
Normal (18.5-24.9)	2 876 759 (46.5)	607 524 (58.5)	935 956 (49.1)	832 275 (42.9)	501 004 (38.5)
Overweight (25.0-29.9)	1 565 519 (25.3)	232 759 (22.4)	479 907 (25.2)	512 325 (26.4)	340 528 (26.1)
Obesity (≥30)	1 513 478 (24.5)	156 286 (15.0)	420 534 (22.1)	524 708 (27.1)	411 950 (31.6)
Missing	356 178	48 680	112 660	121 047	73 791
Nontransfusion SMM					
Obstetric comorbidity index score, median (IQR)	0 (0-7)	1 (0-6)	0 (0-7)	0 (0-7)	1 (0-9)
During delivery hospitalization	45 051 (0.7)	6873 (0.6)	13 005 (0.6)	13 559 (0.7)	11 614 (0.8)
After discharge within 42 d after delivery	13 534 (0.2)	1601 (0.1)	3334 (0.2)	4105 (0.2)	4494 (0.3)
After discharge within 365 d after delivery^a^	26 292 (0.4)	2741 (0.3)	6373 (0.3)	8112 (0.4)	9066 (0.7)
Readmission					
Within 42 d after delivery	73 080 (1.1)	10 035 (0.9)	20 293 (1.0)	22 856 (1.1)	19 896 (1.4)
Within 365 d after delivery^a^	172 192 (3.3)	19 002 (2.3)	44 199 (2.8)	55 806 (3.4)	53 185 (4.7)

Abbreviations: BMI, body mass index (calculated as weight in kilograms divided by height in meters squared); GED, General Educational Development; MVI, Maternal Vulnerability Index; SMM, severe maternal morbidity.

^a^ Data were available for Michigan, Oregon, and South Carolina.

### MVI and Primary Outcomes

**Figure 1.**
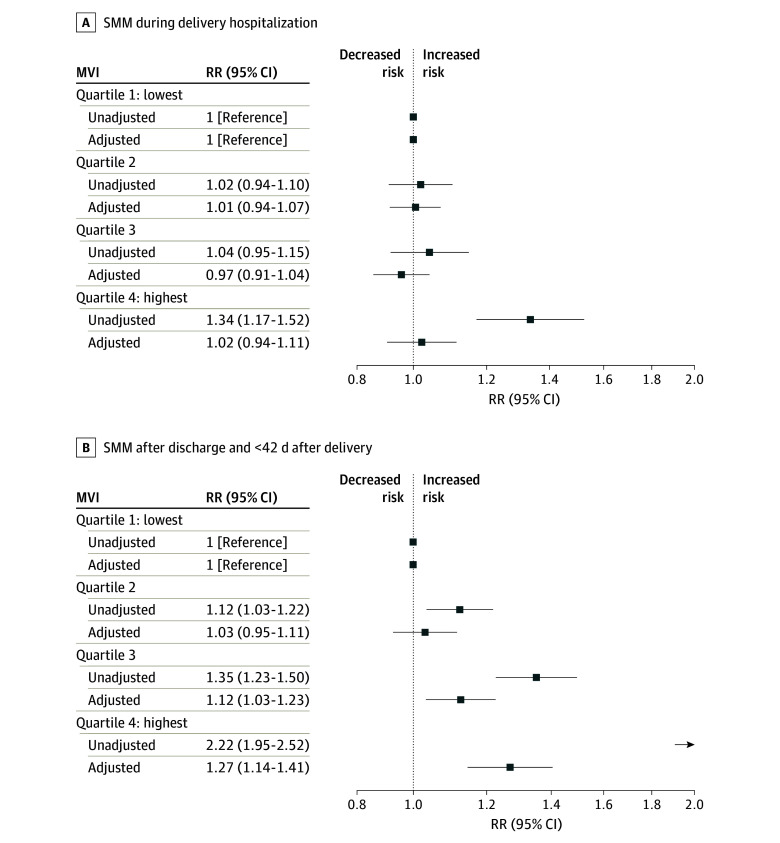
Associations of Maternal Vulnerability Index (MVI) Score With Severe Maternal Morbidity (SMM) Unadjusted and adjusted associations are shown during A, delivery hospitalization and B, after discharge within 42 days after delivery. Models were adjusted for race and ethnicity, insurance type, education, obstetric comorbidity index score, and birth year. RR indicates relative risk.

Figure 1 shows unadjusted and adjusted associations between MVI and SMM during delivery hospitalization and after discharge within 42 days after delivery. In adjusted analyses, no association was found between MVI quartile and SMM during delivery hospitalization. However, a dose-response association was observed between MVI score and SMM after discharge within 42 days after delivery. Specifically, birthing individuals in the second MVI quartile had no significantly increased risk of SMM (aRR, 1.03; 95% CI, 0.95-1.11), those in the third MVI quartile had an increased risk (aRR, 1.12; 95% CI, 1.03-1.23), and those in the fourth or highest MVI quartile experienced an increased risk (aRR, 1.27; 95% CI, 1.14-1.41) compared with individuals in the lowest MVI quartile.

**Figure 2.**
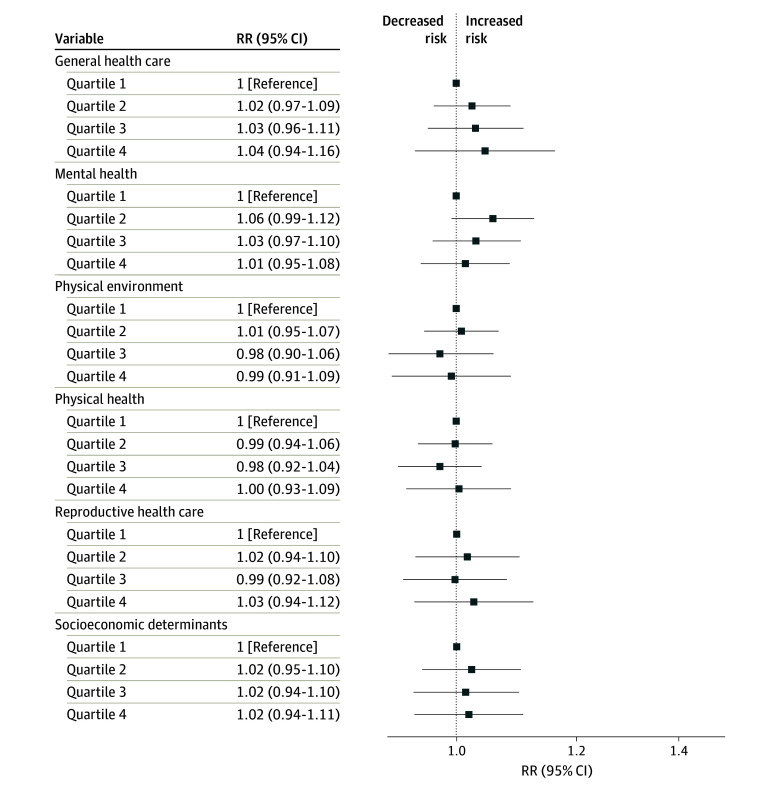
Maternal Vulnerability Index (MVI) Themes and Severe Maternal Morbidity (SMM) During Delivery Hospitalization Models were adjusted for race and ethnicity, insurance type, education, obstetric comorbidity index score, and birth year. RR indicates relative risk.

**Figure 3.**
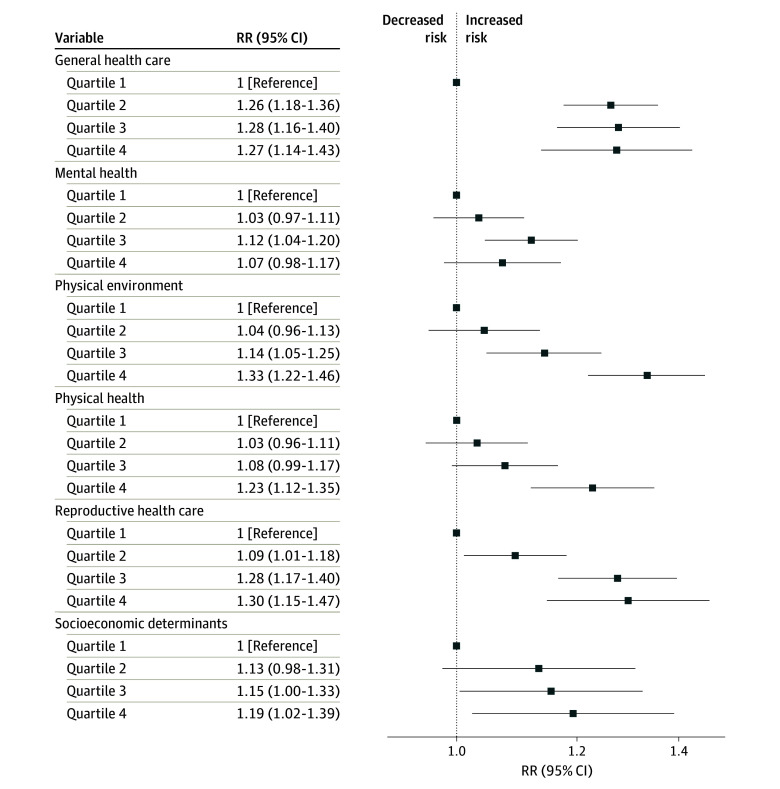
Maternal Vulnerability Index (MVI) Themes and Severe Maternal Morbidity (SMM) After Discharge Within 42 Days After Delivery Models were adjusted for race and ethnicity, insurance type, education, obstetric comorbidity index score, and birth year. RR indicates relative risk.

Unadjusted associations between each MVI theme and the primary outcomes are presented in eTable 3 in Supplement 1, while adjusted associations are presented in Figure 2 and Figure 3. In adjusted analyses, no MVI themes were associated with SMM during delivery hospitalization (Figure 2). However, the highest MVI quartile in the themes of general health care (aRR, 1.27; 95% CI, 1.14-1.43), physical environment (aRR, 1.33; 95% CI, 1.22-1.46), physical health (aRR,1.23; 95% CI, 1.12-1.35), reproductive health care (aRR, 1.30; 95% CI, 1.15-1.47), and socioeconomic determinants (aRR, 1.19; 95% CI, 1.02-1.39) was associated with SMM after discharge within 42 days after delivery (Figure 3). A dose-response association was observed between all MVI themes and SMM after discharge within 42 days after delivery (eg, physical environment MVI theme second quartile: aRR, 1.04; 95% CI, 0.96-1.13; third quartile: aRR, 1.14; 95% CI, 1.05-1.25; fourth quartile: aRR, 1.33; 95% CI, 1.22-1.46), except for the mental health and general health care themes. For mental health, only the third quartile was associated with SMM after discharge within 42 days after delivery (aRR, 1.12; 95% CI, 1.04-1.20). In contrast, for the general health care theme, the second, third, and fourth MVI quartiles showed similar associations with SMM after discharge within 42 days after delivery.

### MVI and Secondary Outcomes

The association of MVI score with SMM during pregnancy, delivery, or within 42 days after delivery and with readmission within 42 days after delivery is presented in eFigure 1 in Supplement 1. Adjusted analyses showed a dose-response association between MVI quartile and both outcomes. Specifically, the highest MVI quartile was associated with SMM during pregnancy, delivery, or within 42 days after delivery (aRR, 1.14; 95% CI, 1.04-1.24) and with readmission within 42 days after delivery (aRR, 1.12; 95% CI, 1.06-1.19).

The association of MVI score with SMM during pregnancy, during delivery, within 365 days after delivery; SMM within 365 days after delivery; and readmission within 365 days after delivery is presented in eFigure 2 in Supplement 1. Analyses showed a dose-response association between MVI quartiles and all outcomes. The highest MVI quartile was associated with SMM during pregnancy, during delivery, or within 365 days after delivery (aRR, 1.19; 95% CI, 1.10-1.27); with SMM within 365 days after delivery (aRR, 1.31; 95% CI, 1.19-1.45); and with readmission within 365 days after delivery (aRR, 1.14; 95% CI, 1.07-1.23).

Sensitivity analysis excluding race and ethnicity from the model yielded consistent results, although the association between higher MVI quartiles and increased risk for SMM was less attenuated compared with the model that adjusted for race and ethnicity (eTable 4 in Supplement 1). Excluding the obstetric comorbidity index from the model did not substantially alter results compared with the fully adjusted model (eTable 4 in Supplement 1). Restricting the study period to 2016 to 2020 also resulted in similar findings (eTable 5 in Supplement 1).

## Discussion

In this cohort study of more than 6.5 million birthing individuals, those residing in ZCTAs with the highest MVI scores experienced an increased adjusted risk (aRR, 1.27; 95% CI, 1.14-1.41) of SMM after discharge within 42 days after delivery compared with those in ZCTAs with the lowest MVI scores. A dose-response association was observed between MVI quartiles and SMM after discharge within 42 days after delivery across themes of reproductive health care, physical health, socioeconomic determinants, and physical environment. Notably, no associations were observed between MVI score or its themes and SMM during delivery hospitalization.

The MVI is a novel tool used to evaluate factors associated with increased risk of adverse health outcomes for birthing individuals across the childbearing continuum. To date, 3 studies have examined the association between MVI, evaluated at the maternal residence county level, and adverse health outcomes. One study^20^ found that residing in counties within the highest MVI quartile compared with the lowest was associated with higher odds of maternal mortality (adjusted odds ratio [aOR], 1.43; 95% CI, 1.20-1.71), low birthweight (aOR, 1.39; 95% CI, 1.37-1.41), and preterm birth (aOR, 1.41; 95% CI, 1.39-1.43) after adjusting for age, educational attainment, and race and ethnicity. Another study^21^ reported that living in the highest vs the lowest MVI quintile was associated with increased odds of preterm birth (aOR, 1.07; 95% CI, 1.01-1.13), with the greatest increase observed for extreme preterm birth (aOR, 1.18; 95% CI, 1.07-1.29) after adjusting for a broader range of variables. A third study^22^ demonstrated that the odds of infant death were higher among birthing individuals residing in counties with the highest MVI scores, with an aOR per 20-point increment in MVI score of 1.06 (95% CI, 1.04-1.07). Our study builds on these findings by highlighting the MVI’s utility in exploring the association between a range of contextual factors and SMM.

Most previous studies examining neighborhood- or county-level factors associated with SMM have focused on single measures, which fail to capture the complex and multidimensional nature of factors associated with maternal and infant health.^4,6,12,13,14,15^ However, 4 studies have explored the association of composite measures of neighborhood social determinants with SMM. In 2 studies, 1 involving Medicaid-insured birthing individuals in Michigan^19^ and another using data from the largest statewide referral hospital in Alabama,^18^ neighborhood deprivation was assessed using the Area Deprivation Index at the census block level. The Alabama study^18^ examined SMM and maternal mortality within 1 year of the delivery encounter and reported increased odds for residents in the highest deprivation neighborhoods, although no adjustments were made for morbidities. Conversely, the Michigan study^19^ found no association between neighborhood deprivation and nontransfusion SMM from delivery up to 6 weeks after delivery, although there was an association with SMM including blood transfusion. In 2 additional studies, 1 from a large safety-net hospital in Georgia^17^ and another population-based study from California,^11^ the association between a composite neighborhood deprivation index (incorporating 8 census tract variables, such as the percentage of adults with less than a high school diploma and the percentage of crowded households) and nontransfusion SMM was examined. The Georgia study^17^ found no association between the deprivation index and SMM recorded at delivery or up to 42 days after delivery. In contrast, the California study^11^ found that individuals residing in the most deprived neighborhoods had higher odds of nontransfusion SMM (aOR, 1.11; 95% CI, 1.08-1.15) during delivery hospitalization compared with those in the least deprived neighborhoods. These findings underscore the need for comprehensive measures that account for the multifaceted nature of neighborhood factors associated with maternal health.

To our knowledge, our study is the first to examine the association between MVI score and SMM. It is noteworthy, although not surprising, that MVI score did not show an association with SMM during delivery hospitalization but was associated with increased SMM in the postpartum period. During delivery hospitalization, birthing individuals typically receive intensive medical care and monitoring in a controlled hospital environment, which can mitigate risk factors. However, in the postpartum period, disparities in access to follow-up care, support services, and living conditions may be associated with increased SMM rates among higher-risk populations. We have previously shown that Black and American Indian individuals, those with lower educational attainment, and individuals with government insurance experience the highest rates of postpartum SMM.^28^ For instance, among White individuals, those with less than a high school diploma were 1.2 times more likely to experience nontransfusion SMM during delivery hospitalization compared with those with at least a college degree.^28^ This disparity increased to 2.6-fold during the postpartum period.^28^ This underscores how socioeconomic factors are associated with exacerbated risk of SMM in the postpartum period. In our study, socioeconomic determinants and physical environment MVI themes were associated with postpartum SMM. Given these findings, it will be critical to examine whether postpartum Medicaid extensions have enhanced the use of outpatient health care during the postpartum period and improved postpartum maternal outcomes. It may also be essential to develop innovative strategies to facilitate access to and use of postpartum care to reach birthing individuals with the highest risk. Promising strategies include telehealth services,^29^ postpartum care navigation programs,^30^ remote blood pressure monitoring,^31,32,33^ and integration of postpartum care into infant well-child visits or the period when the infant is in the neonatal intensive care unit.^34,35^

### Strengths and Limitations

Strengths of this study include examining data from 5 states and adjusting for a wide range of factors. However, there are limitations to consider. The MVI relies on publicly available datasets and may not capture state-specific programs, such as the Alliance for Innovation on Maternal Health, a data-driven maternal safety and quality improvement initiative.^36^ Our data span from 2008 to 2020, whereas the MVI primarily uses data from 2018 through 2021. However, a sensitivity analysis restricting the study period to the general time when the MVI was developed yielded similar findings. It will be interesting to explore whether the MVI changes over time and how these changes may contribute to adverse maternal outcomes. Additionally, we did not have data on geocoded addresses and instead examined the MVI at the ZCTA level.

## Conclusions

In this cohort study, we found that MVI score was not associated with SMM during delivery hospitalization but was associated with postpartum SMM. This suggests that the MVI score may capture long-term risks more effectively than acute conditions seen during delivery hospitalization. These findings suggest that greater investment in high-risk communities and comprehensive maternal care may help reduce postpartum SMM, particularly through enhanced access to maternal health services, community health programs, and telehealth services.

## Notes

Supplement 1. **eTable 1.** Themes, Sub-themes, and Associated Indicators That Comprise the US Maternal Vulnerability Index **eTable 2.** Obstetric Comorbidity Index Variables and Associated Scores for Non-transfusion SMM **eTable 3.** Unadjusted Associations of Maternal Vulnerability Index (MVI) Themes With SMM During Delivery Hospitalization and SMM Within 42 days Postpartum **eTable 4.** Unadjusted and Adjusted Associations of Maternal Vulnerability Index (MVI) With SMM **eTable 5.** Unadjusted and Adjusted Associations of Maternal Vulnerability Index (MVI) With SMM Restricting Data to 2016-2020 **eFigure 1.** Unadjusted and Adjusted Associations of Maternal Vulnerability Index (MVI) With A) SMM During Pregnancy, Delivery, or Within 42 Days Postpartum and B) Readmission Within 42 Days Postpartum **eFigure 2.** Unadjusted and Adjusted Associations of Maternal Vulnerability Index (MVI) With A) SMM During Pregnancy, Delivery, or Within 365 Days Postpartum, B) SMM Post-Discharge Within 365 Days Postpartum, and C) Readmission Within 365 Days Postpartum

Supplement 2.
Data Sharing Statement
