# Supplementary material for: Maternal Vulnerability Index and Severe Maternal Morbidity
Source: JAMA Netw Open. 2025 Jun 23;8(6):e2517068. doi: 10.1001/jamanetworkopen.2025.17068 (PMC12186125; doi:10.1001/jamanetworkopen.2025.17068)
Supplement: Supplement 1. — eTable 1. Themes, Sub-themes, and Associated Indicators That Comprise the US Maternal Vulnerability Index eTable 2. Obstetric Comorbidity Index Variables and Associated Scores for Non-transfusion SMM eTable 3. Unadjusted Associations of Maternal Vulnerability Index (MVI) Themes With SMM During Delivery Hospitalization and SMM Within 42 days Postpartum eTable 4. Unadjusted and Adjusted Associations of Maternal Vulnerability Index (MVI) With SMM eTable 5. Unadjusted and Adjusted Associations of Maternal Vulnerability Index (MVI) With SMM Restricting Data to 2016-2020 eFigure 1. Unadjusted and Adjusted Associations of Maternal Vulnerability Index (MVI) With A) SMM During Pregnancy, Delivery, or Within 42 Days Postpartum and B) Readmission Within 42 Days Postpartum eFigure 2. Unadjusted and Adjusted Associations of Maternal Vulnerability Index (MVI) With A) SMM During Pregnancy, Delivery, or Within 365 Days Postpartum, B) SMM Post-Discharge Within 365 Days Postpartum, and C) Readmission Within 365 Days Postpartum [file jamanetwopen-e2517068-s001.pdf]

Supplemental Online Content

Boghossian NA, Radack J, Passarella M, et al. Maternal Vulnerability Index and severe maternal morbidity. *JAMA Netw Open*. 2025;8(6):e2517068. doi:10.1001/jamanetworkopen.2025.17068

eTable 1. Themes, Sub-themes, and Associated Indicators That Comprise the US Maternal Vulnerability Index

eTable 2. Obstetric Comorbidity Index Variables and Associated Scores for Non-transfusion SMM

eTable 3. Unadjusted Associations of Maternal Vulnerability Index (MVI) Themes With SMM During Delivery Hospitalization and SMM Within 42 days Postpartum

eTable 4. Unadjusted and Adjusted Associations of Maternal Vulnerability Index (MVI) With SMM

eTable 5. Unadjusted and Adjusted Associations of Maternal Vulnerability Index (MVI) With SMM Restricting Data to 2016-2020

eFigure 1. Unadjusted and Adjusted Associations of Maternal Vulnerability Index (MVI) With A) SMM During Pregnancy, Delivery, or Within 42 Days Postpartum and B) Readmission Within 42 Days Postpartum

eFigure 2. Unadjusted and Adjusted Associations of Maternal Vulnerability Index (MVI) With A) SMM During Pregnancy, Delivery, or Within 365 Days Postpartum, B) SMM Post-Discharge Within 365 Days Postpartum, and C) Readmission Within 365 Days Postpartum

This supplemental material has been provided by the authors to give readers additional information about their work.

© 2025 Boghossian NA et al. *JAMA Network Open*.

eTable 1. Themes, Sub-themes, and Associated Indicators That Comprise the US Maternal Vulnerability Index.

Theme	Sub-theme	Indicator
**1. Reproductive Healthcare**	Family planning needs	Female contraceptive clients served at publicly funded clinics per 100,000 women of reproductive age (13-44 years)
	Access to abortions	Minimum distance to nearest abortion clinic for women of reproductive age
		Count of supportive abortion policies
	Public reproductive health funding	Public expenditures for family planning client services per capita
	Reproductive health system capacity	Ratio of OBGYN providers per women of reproductive age
		Ratio of nurse midwives per women of reproductive age
		Ratio of newborn bassinets per women of reproductive age
**2. Physical Health**	Hypertension prevalence	Percent of adult population with high blood pressure
	Diabetes prevalence	Percent of female adult population with diabetes (>20 years by county and >18 years by state)
	Obesity prevalence	Percent of female adult population that is obese (age >20)
	STI prevalence	Prevalence of gonorrhea
		Prevalence of primary or secondary syphilis
		Prevalence of chlamydia
		Prevalence of hepatitis B
	HIV prevalence	Prevalence of HIV in the female population (≥13 years)
	Self-rated health	Percent of the adult population reporting poor or fair health
**3. Mental Health and Substance Abuse**	Stress	Percent of adult population reporting frequent mental distress
		Percent of female worker population with a long commute (>30 minutes)
	Mental health status	Percent of adult population reporting any mental illness
		Percent of adult population ever diagnosed with depression
	Mental health service accessibility	Mental health providers per capita
	Substance abuse	Percent of adults that smoke
		Age adjusted overdose death rate
**4. General Healthcare**	Affordability	Postpartum extension status
		Income eligibility limit for pregnant individuals
		Medicaid expansion status
		Percent of women of reproductive age who are uninsured (19-44 years)
	Accessibility	Distance to the nearest hospital
	Care seeking behavior	Percent of the adult population that reported a routine physical checkup
		Percent of the adult population that reported having a primary care physician
	Quality	Prevention Quality Indicator
**5. Socioeconomic Determinants**	Educational attainment	Percent of women of reproductive age (≥25 years) with a bachelor's degree
		Percent of women of reproductive age (18-44 years) with no high school degree
	Minority status	Percent of the population that speaks English less than well
	Poverty	Percent of women of reproductive age (15-44 years) that live under poverty

© 2025 Boghossian NA et al. *JAMA Network Open*.

Theme	Sub-theme	Indicator
**5. Socioeconomic Determinants**	Food insecurity	Percent of the general population suffering from food insecurity
	Social Capital	Percent of households that are single female headed
		Social capital index
**6. Physical Environment**	Housing	Percent of households with severe housing problems
	Violence	Violent crime rate per 100,000 population
	Transportation	Percent of adult female population (≥16 years) with no access to a vehicle
		Transit Connectivity Index (TCI)
	Air pollution	Concentration of particulate matter of size ≤2.5 micrometers

Adapted from Surgo Ventures (2021). The US Maternal Vulnerability Index (MVI) Methodology.

© 2025 Boghossian NA et al. *JAMA Network Open*.

eTable 2. Obstetric Comorbidity Index Variables and Associated Scores for Non-transfusion SMM

**Comorbidity**	**Score for non-transfusion SMM**
Placenta accreta spectrum	43
Pulmonary hypertension	33
Chronic renal disease	30
Cardiac disease, preexisting	25
HIV/AIDS	13
Preeclampsia with severe features	18
Placental abruption	13
Bleeding disorder, preexisting	11
Anemia, preexisting	6
Twin/multiple pregnancy	5
Preterm birth (< 37 weeks)	13
Placenta previa, complete or partial	3
Neuromuscular disease	13
Asthma, acute or moderate/severe	12
Preeclampsia without severe features or gestational hypertension	7
Connective tissue or autoimmune disease	8
Uterine fibroids	7
Substance use disorder	4
Gastrointestinal disease	7
Chronic hypertension	6
Major mental health disorder	4
Preexisting diabetes mellitus	6
Thyrotoxicosis	2
Previous cesarean birth	0
Gestational diabetes mellitus	2
Delivery BMI ≥ 40	0
Maternal age ≥ 35 years	1

© 2025 Boghossian NA et al. *JAMA Network Open*.

eTable 3. Unadjusted Associations of Maternal Vulnerability Index (MVI) Themes With SMM During Delivery Hospitalization and SMM Within 42 days Postpartum

		Unadjusted RR (95% CI)	
		**SMM during delivery hospitalization**	**SMM within 42 days postpartum**
General Healthcare Quartile	1	Ref	Ref
	2	**1.09 (1.00-1.19)**	**1.34 (1.21-1.49)**
	3	0.97 (0.87-1.10)	1.07 (0.95-1.20)
	4	0.98 (0.84-1.14)	1.06 (0.91-1.22)
Mental Health Quartile	1	Ref	Ref
	2	**1.10 (1.02-1.19)**	**1.28 (1.17-1.41)**
	3	**1.20 (1.08-1.33)**	**1.74 (1.57-1.94)**
	4	**1.26 (1.11-1.43)**	**1.91 (1.70-2.15)**
Physical Environment Quartile	1	Ref	Ref
	2	1.06 (0.99-1.13)	**1.13 (1.03-1.24)**
	3	1.11 (0.99-1.22)	**1.34 (1.20-1.50)**
	4	**1.18 (1.04-1.34)**	**1.84 (1.62-2.10)**
Physical Health Quartile	1	Ref	Ref
	2	1.01 (0.95-1.09)	**1.09 (1.01-1.18)**
	3	1.03 (0.95-1.12)	**1.22 (1.12-1.34)**
	4	**1.32 (1.18-1.48)**	**2.05 (1.83-2.31)**
Reproductive Healthcare Quartile	1	Ref	Ref
	2	1.08 (0.96-1.21)	**1.27 (1.13-1.43)**
	3	1.07 (0.94-1.23)	**1.64 (1.45-1.87)**
	4	1.04 (0.89-1.21)	**1.65 (1.38-1.98)**
Socioeconomic Quartile	1	Ref	Ref
	2	1.07 (0.98-1.16)	1.11 (0.96-1.30)
	3	1.08 (0.99-1.17)	**1.20 (1.04-1.39)**
	4	**1.21 (1.08-1.34)**	**1.48 (1.26-1.74)**

Models adjusted for race/ethnicity, insurance type, education, obstetric comorbidity index score, and birth year.
Bolded figures indicate statistical significance.

© 2025 Boghossian NA et al. *JAMA Network Open*.

eTable 4. Unadjusted and Adjusted Associations of Maternal Vulnerability Index (MVI) With SMM

		Unadjusted RR (95% CI)	Adjusted RR (95% CI)	Adjusted excluding race/ethnicity RR (95% CI)	Adjusted excluding obstetric comorbidity index RR (95% CI)
**SMM during delivery hospitalization**					
MVI Quartile	1	Ref	Ref	Ref	Ref
	2	1.02 (0.94-1.10)	1.01 (0.94-1.07)	1.00 (0.94-1.07)	1.01 (0.94-1.09)
	3	1.04 (0.95-1.15)	0.97 (0.91-1.04)	0.98 (0.91-1.05)	0.99 (0.91-1.08)
	4	**1.34 (1.17-1.52)**	1.02 (0.94-1.11)	1.08 (0.99-1.17)	1.06 (0.96-1.18)
**SMM within 42 days postpartum**					
MVI Quartile	1	Ref	Ref	Ref	Ref
	2	**1.12 (1.03-1.22)**	1.03 (0.95-1.11)	1.03 (0.95-1.12)	1.03 (0.95-1.11)
	3	**1.35 (1.23-1.50)**	**1.12 (1.03-1.23)**	**1.18 (1.08-1.29)**	**1.13 (1.03-1.23)**
	4	**2.22 (1.95-2.52)**	**1.27 (1.14-1.41)**	**1.68 (1.50-1.87)**	**1.29 (1.16-1.44)**
**SMM during pregnancy, delivery, or within 42 days postpartum[1]**					
MVI Quartile	1	Ref	Ref	Ref	Ref
	2	1.09 (0.99-1.20)	1.03 (0.96-1.11)	1.03 (0.95-1.11)	1.04 (0.95-1.14)
	3	**1.22 (1.09-1.38)**	1.06 (0.97-1.15)	1.08 (0.98-1.18)	1.08 (0.97-1.20)
	4	**1.66 (1.43-1.93)**	**1.14 (1.04-1.24)**	**1.29 (1.17-1.43)**	**1.16 (1.02-1.30)**
**SMM during pregnancy, delivery, or within 365 days postpartum[1]**					
MVI Quartile	1	Ref	Ref	Ref	Ref
	2	**1.14 (1.06-1.23)**	1.05 (0.99-1.11)	1.05 (0.99-1.12)	1.06 (0.99-1.14)
	3	**1.30 (1.18-1.43)**	**1.09 (1.01-1.16)**	**1.11 (1.03-1.19)**	**1.11 (1.01-1.21)**
	4	**1.79 (1.58-2.03)**	**1.19 (1.10-1.27)**	**1.34 (1.23-1.45)**	**1.20 (1.09-1.33)**
**SMM within 365 days postpartum[1]**					
MVI Quartile	1	Ref	Ref	Ref	Ref
	2	**1.20 (1.09-1.33)**	1.04 (0.96-1.13)	1.06 (0.98-1.15)	1.05 (0.97-1.14)
	3	**1.53 (1.37-1.71)**	**1.15 (1.04-1.26)**	**1.20 (1.09-1.33)**	**1.16 (1.06-1.27)**
	4	**2.38 (2.10-2.70)**	**1.31 (1.19-1.45)**	**1.64 (1.48-1.82)**	**1.33 (1.20-1.47)**
**Readmission within 42 days postpartum**					
MVI Quartile	1	Ref	Ref	Ref	Ref
	2	**1.09 (1.04-1.14)**	1.02 (0.98-1.06)	1.03 (0.99-1.07)	1.02 (0.98-1.06)
	3	**1.20 (1.14-1.28)**	1.05 (0.99-1.10)	**1.09 (1.03-1.14)**	1.05 (0.99-1.10)
	4	**1.57 (1.45-1.70)**	**1.12 (1.06-1.19)**	**1.30 (1.22-1.39)**	**1.14 (1.07-1.21)**
**Readmission within 365 days postpartum[1]**					
MVI Quartile	1	Ref	Ref	Ref	
	2	**1.10 (1.05-1.17)**	0.98 (0.93-1.03)	0.99 (0.94-1.04)	0.98 (0.94-1.04)
	3	**1.32 (1.24-1.41)**	1.04 (0.98-1.11)	1.06 (0.99-1.14)	1.05 (0.98-1.12)
	4	**1.77 (1.64-1.91)**	**1.14 (1.07-1.23)**	**1.27 (1.18-1.37)**	**1.15 (1.07-1.24)**

Models adjusted for race/ethnicity, insurance type, education, obstetric comorbidity index score, and birth year.
Bolded figures indicate statistical significance.
[1]Data available for Michigan, Oregon, and South Carolina.

© 2025 Boghossian NA et al. *JAMA Network Open*.

eTable 5. Unadjusted and Adjusted Associations of Maternal Vulnerability Index (MVI) With SMM Restricting Data to 2016-2020.

		Unadjusted RR (95% CI)	Adjusted RR (95% CI)	Adjusted excluding race/ethnicity RR (95% CI)	Adjusted excluding obstetric comorbidity index RR (95% CI)
**SMM during delivery hospitalization**					
MVI Quartile	1	Ref	Ref	Ref	
	2	**1.12 (1.00-1.25)**	1.04 (0.96-1.13)	1.03 (0.94-1.12)	1.09 (0.98-1.21)
	3	**1.22 (1.07-1.39)**	1.05 (0.95-1.15)	1.04 (0.95-1.15)	1.10 (0.98-1.24)
	4	**1.48 (1.26-1.74)**	1.05 (0.95-1.16)	1.09 (0.98-1.21)	1.09 (0.95-1.26)
**SMM within 42 days postpartum**					
MVI Quartile	1	Ref	Ref	Ref	
	2	1.21 (0.97-1.52)	1.08 (0.87-1.34)	1.09 (0.87-1.35)	1.09 (0.88-1.36)
	3	**1.57 (1.24-1.99)**	1.22 (0.98-1.53)	**1.30 (1.04-1.62)**	1.24 (0.99-1.56)
	4	**2.60 (2.01-3.36)**	**1.48 (1.17-1.88)**	**1.92 (1.51-2.44)**	**1.50 (1.19-1.90)**
**SMM during pregnancy, delivery, or within 42 days postpartum[1]**					
MVI Quartile	1	Ref	Ref	Ref	
	2	1.10 (0.97-1.24)	1.00 (0.91-1.10)	0.99 (0.90-1.10)	1.03 (0.92-1.16)
	3	**1.25 (1.08-1.44)**	1.03 (0.92-1.15)	1.05 (0.93-1.17)	1.05 (0.92-1.20)
	4	**1.65 (1.39-1.96)**	1.09 (0.98-1.21)	**1.22 (1.09-1.37)**	1.08 (0.94-1.25)
**SMM during pregnancy, delivery, or within 365 days postpartum[1]**					
MVI Quartile	1	Ref	Ref	Ref	
	2	**1.15 (1.03-1.28)**	1.02 (0.94-1.12)	1.02 (0.94-1.12)	1.05 (0.94-1.16)
	3	**1.33 (1.17-1.50)**	1.06 (0.96-1.17)	1.08 (0.97-1.19)	1.08 (0.96-1.21)
	4	**1.80 (1.54-2.09)**	**1.14 (1.03-1.25)**	**1.28 (1.15-1.42)**	1.13 (0.99-1.29)
**SMM within 365 days postpartum[1]**					
MVI Quartile	1	Ref	Ref	Ref	
	2	**1.20 (1.03-1.39)**	1.02 (0.89-1.18)	1.04 (0.90-1.19)	1.03 (0.89-1.19)
	3	**1.49 (1.27-1.75)**	1.08 (0.92-1.26)	1.14 (0.98-1.33)	1.09 (0.94-1.27)
	4	**2.30 (1.93-2.75)**	**1.23 (1.05-1.43)**	**1.53 (1.30-1.80)**	**1.22 (1.04-1.43)**
**Readmission within 42 days postpartum**					
MVI Quartile	1	Ref	Ref	Ref	
	2	**1.23 (1.05-1.43)**	1.14 (0.99-1.29)	**1.14 (1.00-1.30)**	**1.15 (1.01-1.31)**
	3	**1.38 (1.15-1.66)**	**1.18 (1.02-1.37)**	**1.23 (1.06-1.43)**	**1.20 (1.02-1.40)**
	4	**1.90 (1.53-2.35)**	**1.32 (1.12-1.55)**	**1.58 (1.33-1.87)**	**1.33 (1.13-1.57)**
**Readmission within 365 days postpartum[1]**					
MVI Quartile	1	Ref	Ref	Ref	
	2	**1.11 (1.02-1.21)**	0.98 (0.90-1.06)	0.99 (0.91-1.07)	0.99 (0.91-1.07)
	3	**1.34 (1.21-1.48)**	1.04 (0.94-1.15)	1.07 (0.97-1.18)	1.04 (0.94-1.16)
	4	**1.76 (1.55-1.99)**	1.11 (0.99-1.23)	**1.27 (1.13-1.42)**	1.10 (0.99-1.23)

Models adjusted for race/ethnicity, insurance type, education, obstetric comorbidity index score, and birth year.
Bolded figures indicate statistical significance.
[1]Data available for Michigan, Oregon, and South Carolina.

© 2025 Boghossian NA et al. *JAMA Network Open*.

eFigure 1A

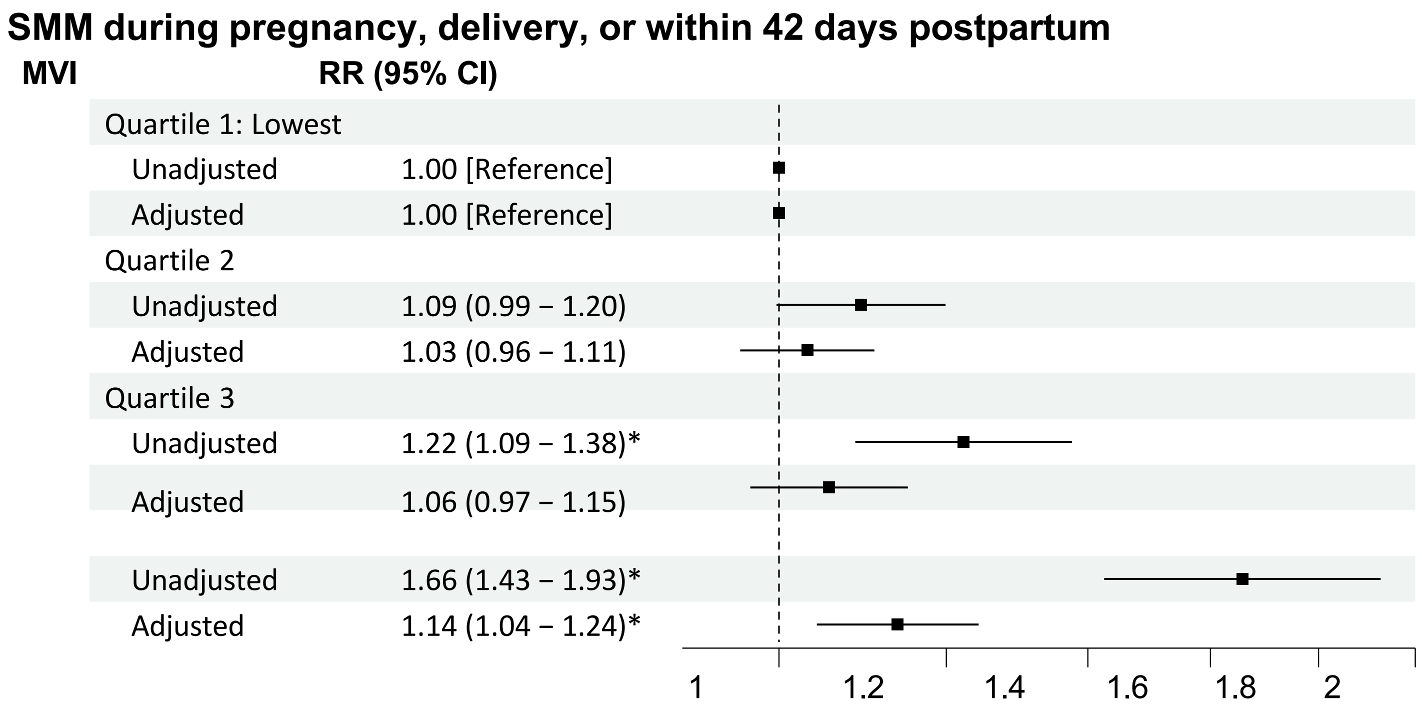

© 2025 Boghossian NA et al. *JAMA Network Open*.

eFigure 1B

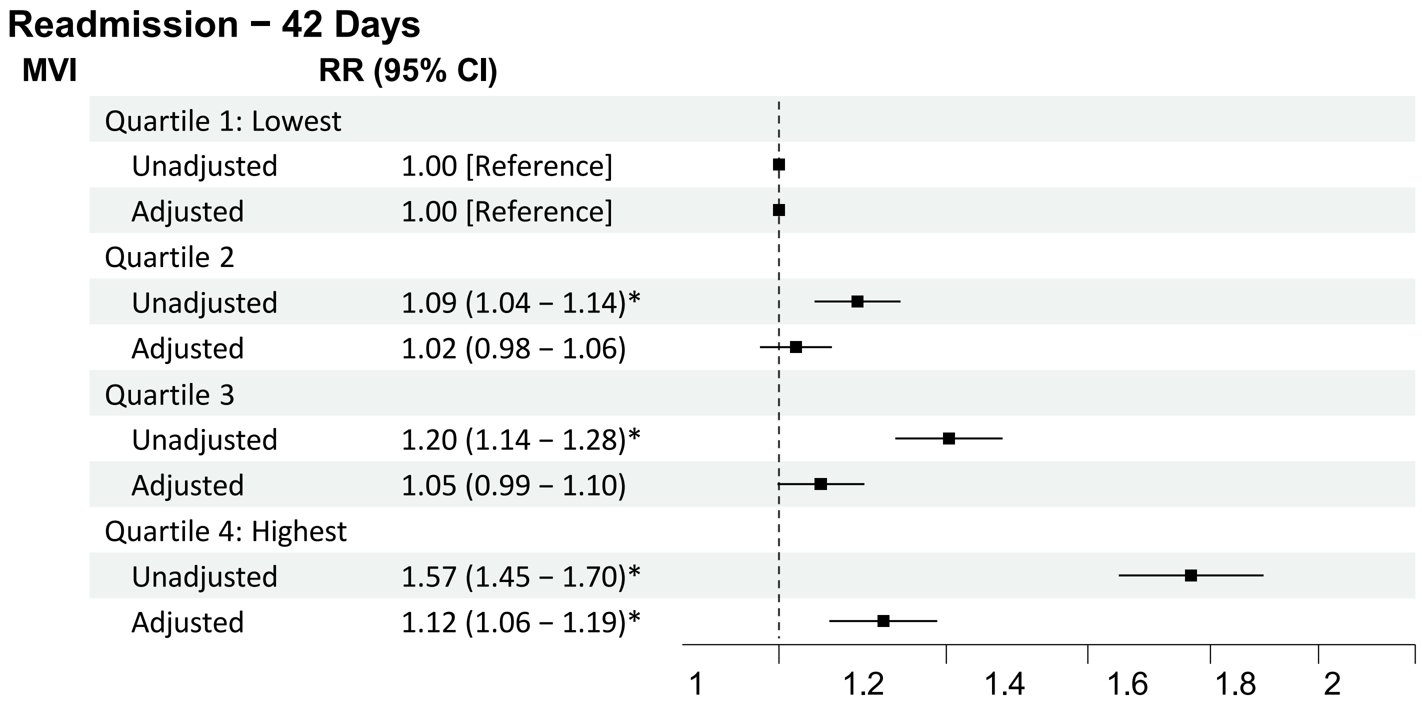

© 2025 Boghossian NA et al. *JAMA Network Open*.

eFigure 2A

SMM during pregnancy, delivery, or within 365 days postpartum

MVI	RR (95% CI)
Quartile 1: Lowest	
Unadjusted	1.00 [Reference]
Adjusted	1.00 [Reference]
Quartile 2	
Unadjusted	1.14 (1.06 – 1.23)*
Adjusted	1.05 (0.99 – 1.11)
Quartile 3	
Unadjusted	1.30 (1.18 – 1.43)*
Adjusted	1.09 (1.01 – 1.16)*
Quartile 4: Highest	
Unadjusted	1.79 (1.58 – 2.03)*
Adjusted	1.19 (1.10 – 1.27)*

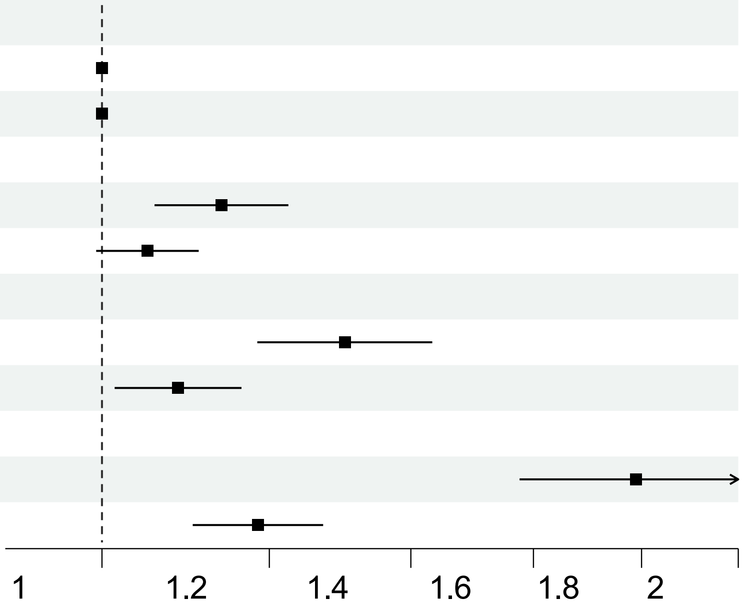

© 2025 Boghossian NA et al. *JAMA Network Open*.

eFigure 2B

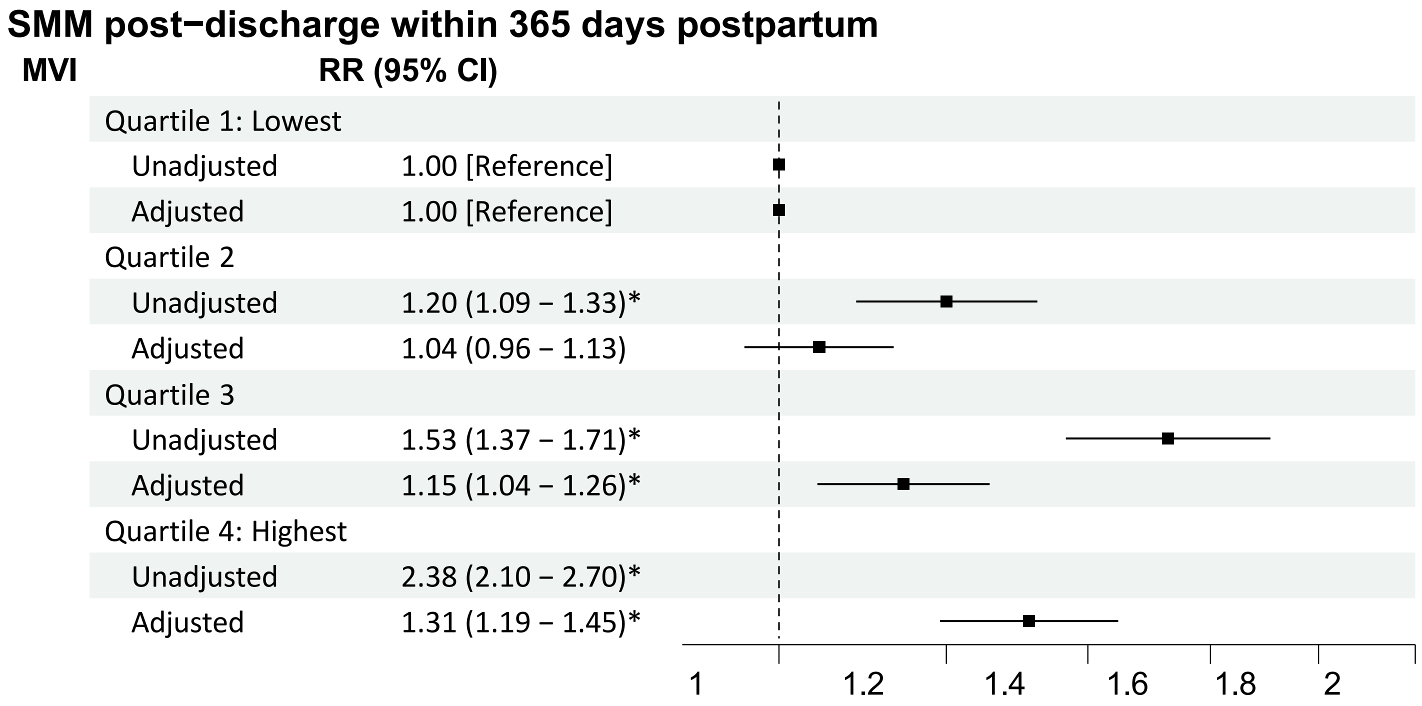

© 2025 Boghossian NA et al. *JAMA Network Open*.

eFigure 2C

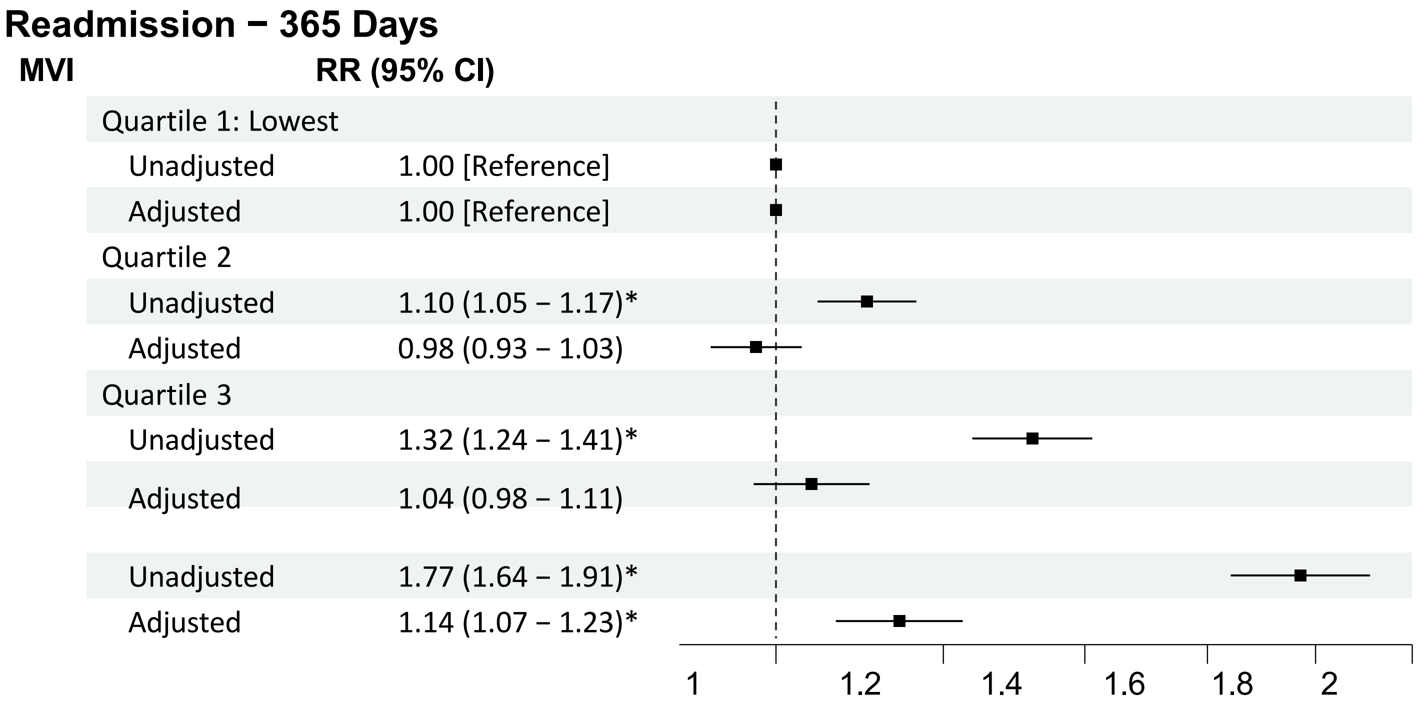

© 2025 Boghossian NA et al. *JAMA Network Open*.
