# Supplementary material for: Maternal Vulnerability Index and Severe Maternal Morbidity
Source: JAMA Netw Open. 2025 Jun 23;8(6):e2517068. doi: 10.1001/jamanetworkopen.2025.17068 (PMC12186125; doi:10.1001/jamanetworkopen.2025.17068)
Supplement: Supplement 2. — Data Sharing Statement [file jamanetwopen-e2517068-s002.pdf]

Data Sharing Statement

Boghossian. Maternal Vulnerability Index and Severe Maternal Morbidity. *JAMA Netw Open*. Published online June 23, 2025. doi:10.1001/jamanetworkopen.2025.17068

Data
Data available: No

Additional Information
Explanation for why data not available: The authors cannot provide access to the data due to Data Use Agreements with each state.
